# Serum Malondialdehyde-Modified Low-Density Lipoprotein Is a Risk Factor for Central Arterial Stiffness in Maintenance Hemodialysis Patients

**DOI:** 10.3390/nu12072160

**Published:** 2020-07-21

**Authors:** Jia-Sian Hou, Chih-Hsien Wang, Yu-Hsien Lai, Chiu-Huang Kuo, Yu-Li Lin, Bang-Gee Hsu, Jen-Pi Tsai

**Affiliations:** 1 Division of Nephrology, Hualien Tzu Chi Hospital, Buddhist Tzu Chi Medical Foundation, Hualien 97010, Taiwan; simianlkive@gmail.com (J.-S.H.); wangch33@gmail.com (C.-H.W.); hsienhsien@gmail.com (Y.-H.L.); hermit.kuo@gmail.com (C.-H.K.); nomo8931126@gmail.com (Y.-L.L.); 2 School of Medicine, Tzu Chi University, Hualien 97004, Taiwan; 3 School of Post-Baccalaureate Chinese Medicine, Tzu Chi University, Hualien 97010, Taiwan; 4 Division of Nephrology, Department of Internal Medicine, Dalin Tzu Chi Hospital, Buddhist Tzu Chi Medical Foundation, Chiayi 62247, Taiwan

**Keywords:** malondialdehyde-modified low-density lipoprotein, aortic stiffness, hemodialysis, carotid-femoral pulse wave velocity

## Abstract

Circulating malondialdehyde-modified low-density lipoprotein (MDA-LDL) acts as a marker of oxidative stress and is associated with atherosclerotic cardiovascular disease. The relationship between serum MDA-LDL levels and aortic stiffness (AS) in patients with hemodialysis (HD) was evaluated. There were 155 HD patients enrolled in this study. Carotid-femoral pulse wave velocity (cfPWV) was measured by a validated tonometry system. Patients with cfPWV >10 m/s were used to define the AS group, while those with values of ≤10 m/s were regarded as the control group. Serum MDA-LDL levels were measured using a commercial enzyme-linked immunosorbent assay. Sixty-eight patients (43.9%) who were defined as AS sufferers, and were older, had a higher percentage of diabetes and hypertension and higher systolic blood pressure and serum MDA-LDL level compared to subjects in the control group. After adjusting for factors significantly associated with AS by multivariable logistic regression analysis, it was revealed that serum MDA-LDL levels, diabetes, and hypertension were independent predictors of AS in HD patients. Multivariable forward stepwise linear regression analysis also showed that a logarithmically transformed MDA-LDL level was significantly correlated with cfPWV values in HD patients. In HD patients, a high serum MDA-LDL level was positively associated with cfPWV values and was a significant predictor of the development of high AS.

## 1. Introduction

Cardiovascular disease (CVD), for which traditional risk factors include diabetes mellitus (DM) and hypertension (HTN), as well as chronic kidney disease (CKD)-specific risk factors, has long been known as the main cause of adverse long-term outcomes in patients with end-stage renal disease (ESRD) receiving maintenance hemodialysis (HD) [1]. Evidence has shown that the stiffening of vascular walls caused by deregulation of elastin and collagen, oxidative stress, disordered mineral metabolisms, and low-grade inflammation, may result in increased myocardial pre-load, and a decrease in perfusion pressure of the coronary artery and future CVD in CKD patients [2,3]. As arterial stiffening increases, there is abnormal pressure amplification with increased reflection wave and earlier backward wave ascending to aorta during systole, which together leads to high stress on central vasculature, as well as increased pulse pressure exposure to those feeding arteries or low impedance vasculature with the results of high blood pressure and mechanical strain to organ parenchyma and future cardiovascular (CV) events [2,4,5]. Pulse-wave velocity (PWV), which is a way to measure vascular function non-invasively and is a surrogate marker of aortic stiffness (AS), has been considered to be associated strongly with future CV events and mortality in ESRD patients, irrespective of traditional CV risk factors [2,6].

Oxidized low-density lipoprotein (LDL), which is formed through the post-translational oxidative modification of LDL by oxidative stress in atherosclerotic lesions and uptake of macrophages by scavenger receptors, as well as the release of inflammatory cytokines from foam cells, plays an important role in the process of atherosclerosis and has been reported to be associated with brachial-ankle PWV (baPWV) in patients with metabolic syndrome or pre-HTN [7,8]. Titer of autoantibody against specific epitopes of oxidized LDL can be used to measure levels of LDL oxidation and as a biomarker for CV events but is limited by its indirect estimation and the inconclusive prognostic value of coronary artery disease (CAD) [9,10]. Circulating malondialdehyde-modified low-density lipoprotein (MDA-LDL), which is a type of oxidized LDL with malondialdehyde-modified (MDA) adduction onto apolipoprotein B, has been well-known to be linked to atherosclerosis in patients of CAD [11,12], as well as being shown to be a useful predictor for major CV events or vulnerable coronary arterial plaque for CAD in DM patients [13,14,15]. MDA-LDL was additionally reported to be associated negatively with a peak size of LDL, which was associated with the progression of atherosclerosis [12]. In addition, the evidence shows that there is a significant association between MDA-LDL and vascular function measured by baPWV, resistance index, and flow-mediated dilatation using lipid-lowering therapy in hypercholesterolemia patients [16,17], as well as being negatively associated with flow-mediated dilatation of brachial artery [18] and positively associated with the calcification score of the coronary artery in HD patients [19].

Because there is scarce evidence about the relationship between MDA-LDL and AS measured by carotid-femoral PWV (cfPWV) in HD patients, we have conducted this cross-sectional study in HD patients to clarify the association.

## 2. Materials and Methods

### 2.1. Patients

Between March and July 2016, 155 HD patients were enrolled at a medical center in Hualien, Taiwan. Those over 20 years old who had received a standard, 4h session of HD, thrice a week for at least 3 months using standard bicarbonate dialysate were enrolled. All patients received disposable, high-flux, polysulfone artificial kidneys (FX class dialyzer, Fresenius Medical Care, Bad Homburg, Germany). “After being approved by the Research Ethics Committee, Hualien Tzu Chi Hospital, Buddhist Tzu Chi Medical Foundation of this study (IRB103–136-B), we enrolled all HD patients at our hospital except those who had active infection, acute myocardial infarction, stroke, peripheral arterial occlusive disease, or pulmonary edema at the time of blood sampling, or who declined to provide informed consent for this study.”

### 2.2. Anthropometric Measurements

Body weight and height of all HD patients were measured, respectively, by the same operator to the nearest half-kilogram or half-centimeter, with patients wearing light clothing with bare feet. Waist circumference was measured midway between the lower rib margin and the iliac crest. Body mass index (BMI) was calculated as the weight (kilograms) divided by height squared (meters). 

### 2.3. Determinations of Carotid-Femoral Pulse Wave Velocity 

Carotid-femoral PWV (cfPWV) was measured by applanation tonometry (SphygmoCor system, AtCor Medical, West Ryde, Australia), as previous studies have also done [20]. Measurements were performed in a supine position after a minimum of 10 min rest in a quiet and temperature-controlled room. Records were taken simultaneously with electrocardiogram signals, which provided an R-timing reference. Pulse wave recordings were performed consecutively at two points on the superficial arteries (carotid-femoral segment). Integral software was used to process each set of pulse waves and electrocardiogram data to calculate the mean time difference between pulse wave and R-wave on a beat-to-beat basis, with an average of 10 consecutive cardiac cycles. The cfPWV was calculated using the difference in elapsed time and distance between the two recorded artery sites. Quality indices included in the software were set to ensure uniformity of data. A cfPWV > 10 m/s defined the high central arterial stiffness (AS) group and ≤10 m/s defined the control group, according to the European Society of Cardiology and the European Society of Hypertension guidelines [21].

### 2.4. Biochemical Determination

Blood samples were collected before patients received HD. Blood samples (approximately 5 mL) were immediately centrifuged at 3000× *g* for 10 min. The serum samples were stored at 4 °C and used for biochemical analyses within 1 h of collection. Serum values of blood urea nitrogen, creatinine, glucose, total cholesterol, triglyceride, total calcium, and phosphorus were measured using an autoanalyzer (Siemens Advia 1800, Siemens Healthcare GmbH, Henkestr, Germany). The fractional clearance index for urea (Kt/V) and urea reduction ratio were measured before dialysis and immediately afterwards using a formal, single-compartment dialysis urea kinetic model. The serum values of intact parathyroid hormone (iPTH) (Diagnostic Systems Laboratories, Webster, TX, USA) and MDA-LDL (Sekisui Diagnostics GmbH, Kaplaneigasse, Pfungstadt, Germany) were measured using commercially available, enzyme-linked, immunosorbent assays.

### 2.5. Statistical Analysis

Continuous variables were tested for normal distribution by the Kolmogorov–Smirnov test. Data have been expressed as the mean ± standard deviation or median with interquartile range (IQR), depending on normal distribution. Comparisons between the high-AS and control group were performed by the Student’s independent t-test or Mann-Whitney U test (two-tailed), accordingly. Categorical data were analyzed by the χ^2^ test and represented as a number and percentage. Nonnormally distributed continuous variables were logarithmically transformed when applied to linear regression analysis. Multivariate logistic and linear regression analyses were used to analyze the relationship between all variables and cfPWV and the risk factors for developing high AS in HD patients. A receiver operating characteristic (ROC) curve was used to calculate the area under the curve (AUC) to identify a cut-off value of MDA-LDL to predict high AS in HD patients. A *p*-value < 0.05 was considered statistically significant. Data were analyzed by SPSS for Windows (version 19.0; SPSS Inc., Chicago, IL, USA).

## 3. Results

Of the HD patients, 77 (49.7%) were female and the average age was 63.15 ± 13.19 years, with 56.16 months (IQR 23.04–117.84) median duration of receiving HD. Sixty-six (42.6%) and 79 (51.0%) patients had DM and HTN, individually. The adequacy of dialysis presented as Kt/V and urea reduction ratio were 1.34 ± 0.17 and 0.73 ± 0.04, respectively. The values of MDA-LDL of all HD patients were 89.15 mg/dL (IQR 60.80–146.24) (Table 1).

Sixty-eight patients (43.9%) were defined as being in the AS group. These were older (65.63 ± 12.17 vs. 61.22 ± 13.69 years old, *p* = 0.038), had higher percentage of DM (60.3% vs. 28.7%, *p* < 0.001) and HTN (61.8% vs. 42.5%, *p* = 0.017), and higher systolic blood pressure (SBP, 149.88 ± 24.84 vs. 138.72 ± 27.11 mmHg, *p* = 0.009) and MDA-LDL (120.63 [82.75–191.74] vs. 72.65 [57.34–112.37] mg/dL, *p* < 0.001) than the control group (Table 1). There were no statistically significant differences in HD duration, body composition, and serum values of dialysis clearance, lipid profiles, or other clinical characteristics or medication use between these two groups.

After adjusting the factors significantly associated with AS (age, DM, HTN, SBP, and MDA-LDL) in univariate logistic regression analysis, MDA-LDL (odds ratio [OR] 1.014, 95% C.I. 1.007–1.021, *p* < 0.001), DM (OR = 2.893, 95% C.I.: 1.300–6.437, *p* = 0.009) and HTN (OR = 2.408, 95% C.I: 1.066–5.436, *p* = 0.034) were found to be significant independent risk factors for developing high AS by multivariate logistic regression analysis (Table 2).

The results showed that cfPWV was significantly positively correlated with age, DM, HTN, SBP, as well as logarithmically transformed glucose and MDA-LDL by simple linear regression analysis. After being analyzed by multivariate stepwise linear regression analysis, DM (*β* = 0.233, adjusted R^2^ change = 0.055, *p* = 0.001), HTN (*β* = 0.132, adjusted R^2^ change = 0.014, *p* = 0.048), and higher logarithmically transformed MDA-LDL levels (*β* = 0.404, adjusted R^2^ change = 0.265, *p* < 0.001) were significantly correlated with cfPWV (Table 3).

**Figure 1:**
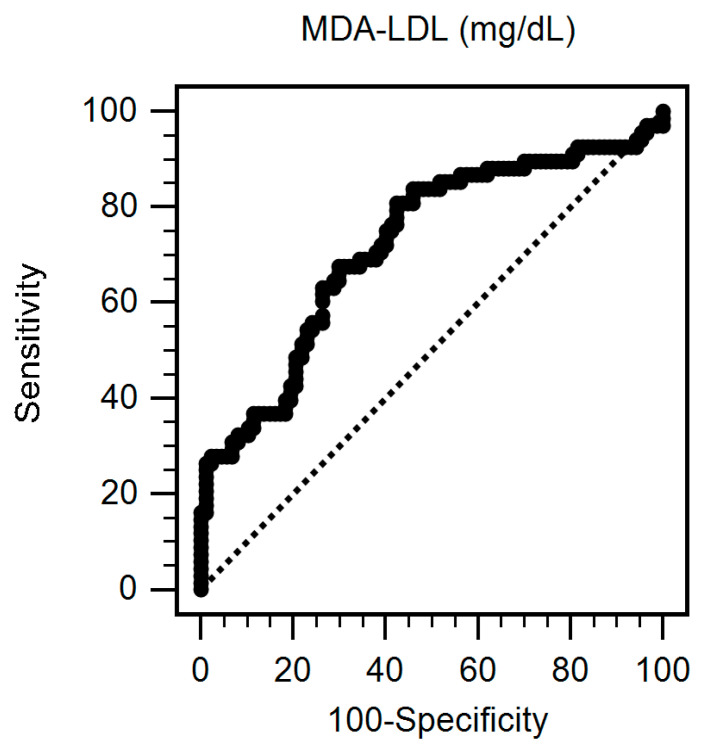
Receiver operating characteristic (ROC) curve analysis to predict aortic stiffness in 155 hemodialysis patients. The area under the ROC curve (AUC) indicates the diagnostic power of malondialdehyde-modified low-density lipoprotein (MDA-LDL) level at predicting aortic stiffness in hemodialysis patients. The AUC for MDA-LDL level was 0.721 (95% confidence interval: 0.643–0.790, *p* < 0.001).

By using the ROC curve to predict AS (Figure 1), it showed that the best cut-off serum value of logarithmically transformed MDA-LDL was 80.33 mg/dL with AUC 0.721 (95% C.I. 0.643–0.790, *p* < 0.001), sensitivity 80.88% (95% C.I. 69.5–89.4%), and specificity 57.47% (95% C.I. 46.4%–68%), respectively.

## 4. Discussion

The major findings of this study are that, in addition to DM and HTN, a high serum MDA-LDL level was associated with high cfPWV values and could predict the development of AS in HD patients.

Arterial stiffness, which was caused by multiple risk factors, resulted in irreversible changes of vascular wall structures with increased pulse pressure to low impedance circulation, along with exposure to high blood pressure (BP) and mechanical strain, which resulted in CVD, renal dysfunction, and mortality [2,3]. In a cross-sectional study of 4336 healthy subjects, there was a progressively and significantly increased baPWV with age [22]. Evidence has shown that aging might additionally induce vascular structural and functional changes in CKD patients, besides abnormal mineral metabolism, with the results of elastin fragmentation and medial layer calcification [2,23]. In ESRD patients, there was marked aging-related AS indicated by an age-associated increase in aortic PWV and decrease in aortic taper, as well as lower brachial/aortic stiffness gradient [23]. Progression of AS reduced the vascular lumen and led to a return of the reflected wave in late systole prematurely, resulting in increased pulse pressure accompanied with increased SBP and decreased DBP [24]. Studies have shown that cfPWV is positively correlated with SBP, along with MetS and waist circumference in HTN and DM patients [25,26]. Moreover, a systemic review that included 26,970 subjects underscored that, in addition to traditional risk factors, such as gender, dyslipidemia, smoking, and BMI, BP elevation and aging were independently associated with cfPWV [27]. In addition, impaired glucose tolerance was independently related to impaired arterial compliance, decreased carotid-femoral transit time, and increased aortic augmentation index in a cross-sectional population study [28]. In DM patients, there was an increase in the production of advanced glycation end products, which showed significant association with cfPWV independent of age, gender, BP, or fasting sugar in a community-dwelling population [29]. Moreover, increased cfPWV was associated with longer DM duration, independent of age, gender, BP, or renal function [30]. Our previous study revealed that CAD patients in the high-AS group had a higher percentage of DM, with higher fasting sugar and serum levels of insulin and HOMA-IR, compared to patients with low AS [31]. One meta-analysis, which enrolled 1222 DM patients, showed a significant association between DM incidence and AS measured by cfPWV [32]. Taken together, age and BP, as well as DM, were consistently and independently associated with cfPWV. Similarly, we found that being older, or having DM or HTN, is associated with a higher degree of cfPWV. Furthermore, we similarly found that DM and HTN are independently significant predictors for the development of high AS, after adjusting the confounders of HD patients.

The onset and acceleration of atherosclerotic lesions is related to oxidative modification, such as the oxygen and peroxyl radical synthesis, of LDL cholesterol [33]. Oxidized LDL is directly chemotactic for monocytes and could modulate the progression of arteriosclerosis after being incorporated via scavenger receptors, which induces monocytes transformed into macrophages and the proliferation of vascular smooth muscle cells, to increase foam cell formation in atherosclerotic lesions, endothelial injury, and plaque formation [34,35]. Oxidized LDL could exert a number of adverse effects on vasculature and CVD [7,8,22,36]. In a longitudinal study of 417 subjects, it was shown that vascular stiffness measured by cfPWV correlated with oxidized LDL in subjects with normal renal function and CKD stage II [36]. In participants who received health exams, a progressively and significantly increased baPWV and plasma oxidized LDL were found in those who were older than 45 years [22]. In addition, circulating oxidized LDL was reported to be associated with baPWV in patients with metabolic syndrome or pre-HTN [7,8].

As a marker of oxidative stress, MDA-LDL is a major end product of LDL oxidation and evidence has shown that MDA-LDL, which reflects endothelial injury or plaque instability, together with troponin I, could discriminate between stable CAD and acute myocardial infarction with high sensitivity and specificity [37]. In addition, MDA-LDL is a predictor for major CV events or vulnerable coronary arterial plaque in coronary artery disease (CAD) and DM patients [13,14,15]. Moreover, MDA-LDL was negatively associated with endothelial function measured by flow-mediated dilatation of the brachial artery [18] and positively correlated to levels of LDL, triglyceride and the severity of albuminuria, which was associated with high AS in the Framingham Study [22,38]. A cross-sectional study of HD patients revealed that there was significant increased baPWV and MDA-LDL, as well as C-reactive protein and reactive oxygen species, in those with intradialytic hypotension, which indicated that AS of this population was related to oxidative stress [39]. The MDA-LDL/LDL ratio, which indicated the extent of MDA modification per LDL particle, was a strong marker for CVD [18] and was significantly associated with coronary artery calcification in HD patients [19]. Lipid-lowering therapy with antioxidant effects has been reportedly associated with decreased values of MDA-LDL and MDA-LDL/LDL ratio, along with improved vascular function measured by baPWV, resistance index, and flow-mediated dilatation in hypercholesterolemia patients [16,17]. However, there was no significant difference in levels of MDA-LDL between those who used medications and those who did not (no statin, 90.88 mg/dL IQR 32.14–447.97 mg/dL; statin, 88.06 mg/dL, IQR 42.59–656.13 mg/dL; *p* = 0.443; data not shown) in this study. Taken together, other than these traditional and CKD-specific risk factors, there might be a role for MDA-LDL in the development of high AS in HD patients, but the definite mechanism and therapies need to be studied.

This study is limited by being cross-sectional and conducted at a single center with a limited number of HD patients. The other limitation was that we only measured MDA-LDL, but evidence had shown that measuring MDA-LDL was correlated negatively with LDL size as well as being located in the dense LDL fraction and could be a useful marker of progression of atherosclerosis in CAD patients [12]. Therefore, the association of serum MDA-LDL or other types of LDL with AS, as well as the role of the development of AS in HD patients, should be confirmed by further longitudinal studies before a cause–effect can be established.

## 5. Conclusions

In addition to DM and HTN, serum MDA-LDL greater than 80.33 mg/dL was shown to be related to AS in HD patients in this study. The positive relationship between MDA-LDL and AS is both promising and provoking, as both were shown to be individually correlated with future CV events. These findings indicate that MDA-LDL may play a role in the pathogenesis of high AS and may also become an alternative therapeutic target to prevent the development of AS in HD patients, but the mechanism and feasibility remain to be further elucidated.

## Figures and Tables

**Table 1:** Clinical variables of the 155 hemodialysis (HD) patients with or without aortic stiffness (AS).

Characteristics	All Patients (*N* = 155)	Control Group (*N* = 87)	Aortic Stiffness Group (*N* = 68)	*p*-Value
Age (years)	63.15 ± 13.19	61.22 ± 13.69	65.63 ± 12.17	0.038 *
Hemodialysis duration (months)	56.16 (23.04–117.84)	69.84 (21.72–134.40)	51.84 (24.99–87.24)	0.241
Height (cm)	159.84 ± 8.54	159.06 ± 8.89	160.85 ± 8.04	0.195
Body weight (Kg)	64.24 ± 15.37	62.94 ± 15.68	65.93 ± 14.91	0.233
Body mass index (Kg/m^2^)	24.95 ± 5.03	24.73 ± 5.23	25.23 ± 4.78	0.544
Carotid-femoral PWV (m/s)	9.88 ± 2.78	7.82 ± 1.22	12.52 ± 1.81	<0.001 *
Systolic blood pressure (mmHg)	143.62 ± 26.64	138.72 ± 27.11	149.88 ± 24.84	0.009 *
Diastolic blood pressure (mmHg)	77.74 ± 16.53	77.46 ± 16.19	78.09 ± 17.08	0.815
Total cholesterol (mg/dL)	144.97 ± 33.82	147.08 ± 37.64	142.26 ± 28.22	0.381
Triglyceride (mg/dL)	117.00 (84.00–187.00)	112.00 (84.00–200.00)	122.50 (87.00–175.75)	0.832
MDA-LDL (mg/dL)	89.15 (60.80–146.24)	72.65 (57.34–112.37)	120.63 (82.75–191.74)	<0.001 *
Albumin (mg/dL)	4.10 (3.90–4.40)	4.10 (3.90–4.40)	4.10 (3.90–4.30)	0.696
Glucose (mg/dL)	131.00 (110.00–169.00)	128.00 (104.00–156.00)	136.00 (114.00–184.00)	0.081
Blood urea nitrogen (mg/dL)	61.30 ± 14.90	61.13 ± 14.20	61.51 ± 15.86	0.873
Creatinine (mg/dL)	9.37 ± 2.08	9.43 ± 2.07	9.30 ± 2.10	0.703
Total calcium (mg/dL)	9.05 ± 0.73	8.98 ± 0.68	9.14 ± 0.79	0.190
Phosphorus (mg/dL)	4.74 ± 1.29	4.73 ± 1.32	4.74 ± 1.26	0.976
Intact parathyroid hormone (pg/mL)	198.00 (69.30–453.80)	244.40 (98.50–453.80)	158.95 (47.83–448.50)	0.129
Urea reduction rate	0.73 ± 0.04	0.74 ± 0.05	0.73 ± 0.04	0.437
Kt/V (Gotch)	1.34 ± 0.17	1.35 ± 0.18	1.32 ± 0.16	0.382
Female, *n* (%)	77 (49.7)	47 (54.0)	30 (44.1)	0.221
Diabetes mellitus, *n* (%)	66 (42.6)	25 (28.7)	41 (60.3)	<0.001 *
Hypertension, *n* (%)	79 (51.0)	37 (42.5)	42 (61.8)	0.017 *
Angiotensin receptor blocker, *n* (%)	44 (28.4)	22 (25.3)	22 (32.4)	0.333
β-blocker, *n* (%)	47 (30.3)	25 (28.7)	22 (32.4)	0.625
Calcium channel blocker, *n* (%)	59 (38.1)	35 (40.2)	24 (35.3)	0.530
Statin, *n* (%)	27 (17.4)	12 (13.8)	15 (22.1)	0.178
Fibrate, *n* (%)	23 (14.8)	13 (14.9)	10 (14.7)	0.967

Values for continuous variables are shown as mean ± standard deviation after analysis by Student’s *t*-test; variables not normally distributed are shown as median and interquartile range after analysis by the Mann-Whitney U test; values are presented as a number (%) and after analysis by the chi-square test. MDA-LDL: malondialdehyde-modified low-density lipoprotein; Kt/V: fractional clearance index for urea; PWV: pulse wave velocity. * *p* < 0.05 was considered statistically significant.

**Table 2:** Multivariate logistic regression analysis of the factors correlated to AS among 155 HD patients.

Variables	Odds Ratio	95% Confidence Interval	*p*-Value
MDA-LDL, 1 mg/dL	1.014	1.007–1.021	<0.001 *
Diabetes mellitus, present	2.893	1.300–6.437	0.009 *
Hypertension, present	2.408	1.066–5.436	0.034 *
Age, 1 year	1.024	0.993–1.055	0.131
Systolic blood pressure, 1 mmHg	1.003	0.988–1.020	0.674

Analysis data was done using the multivariate logistic regression analysis (adopted factors: diabetes mellitus, hypertension, age, systolic blood pressure, and MDA-LDL). MDA-LDL: malondialdehyde-modified low-density lipoprotein. * *p* < 0.05 was considered statistically significant.

**Table 3:** Correlation between central PWV levels and clinical variables among 155 HD patients.

Variables	Central PWV (m/s)				
	Univariate		Multivariate		
	*r*	*p*-Value	Standardized Beta	Adjusted R^2^ Change	*p*-Value
Diabetes mellitus	0.351	<0.001 *	0.233	0.055	0.001 *
Hypertension	0.177	0.028 *	0.132	0.014	0.048 *
Age (years)	0.199	0.013 *	–	–	–
Log-HD duration (months)	−0.122	0.131	–	–	–
Height (cm)	0.171	0.033 *	–	–	–
Body weight (Kg)	0.178	0.027 *	–	–	–
Body mass index (Kg/m^2^)	0.111	0.167	–	–	–
Systolic blood pressure (mmHg)	0.260	0.001 *	–	–	–
Diastolic blood pressure (mmHg)	0.079	0.332	–	–	–
Total cholesterol (mg/dL)	−0.045	0.567	–	–	–
Log-Triglyceride (mg/dL)	0.108	0.180	–	–	–
Log-MDA-LDL (mg/dL)	0.520	<0.001 *	0.404	0.265	<0.001 *
Log-Albumin (mg/dL)	0.099	0.220	–	–	–
Log-Glucose (mg/dL)	0.164	0.042 *	–	–	–
Blood urea nitrogen (mg/dL)	0.042	0.600	–	–	–
Creatinine (mg/dL)	0.076	0.345	–	–	–
Total calcium (mg/dL)	0.103	0.204	–	–	–
Phosphorus (mg/dL)	0.030	0.715	–	–	–
Log-iPTH (pg/mL)	−0.118	0.143	–	–	–
Urea reduction rate	−0.088	0.276	–	–	–
Kt/V (Gotch)	−0.092	0.256	–	–	–

Data of HD duration, triglyceride, glucose, iPTH, and MDA-LDL levels showed skewed distribution, and, therefore, were log-transformed before analysis. Analysis data was done using the univariate linear regression analysis or multivariate stepwise linear regression analysis (adopted factors: diabetes mellitus, hypertension, age, height, body weight, systolic blood pressure, log-glucose, and log-MDA-LDL). HD: hemodialysis; MDA-LDL: malondialdehyde-modified low-density lipoprotein; iPTH: intact parathyroid hormone; Kt/V: fractional clearance index for urea. * *p* < 0.05 was considered statistically significant.
